# Patient's healthcare needs in the traditional and technological neuro-rehabilitation field: a survey methodological approach

**DOI:** 10.3389/fdgth.2026.1638302

**Published:** 2026-02-03

**Authors:** Elena Beani, Alessio Fasano, Maria Cristina Mauro, Marco Germanotta, Monia Andrea Papa, Silvana Quaglini, Gaetano Maria Celardo, Federica Camuncoli, Giovanni Cioni, Francesca Fedeli, Paolo Fogar, Giuseppe Turchetti, Leopoldo Trieste, Christian Cipriani, Giovanni Comandè, Stefano Denicolai, Giuseppina Sgandurra, Irene Giovanna Aprile, The Mission 1 Fit4Med Consortium

**Affiliations:** 1 Department of Developmental Neuroscience, IRCCS Fondazione Stella Maris, Pisa, Italy; 2 Department of Clinical and Experimental Medicine, University of Pisa, Pisa, Italy; 3 IRCCS Fondazione Don Carlo Gnocchi, Florence, Italy; 4 Department of Electrical, Computer and Biomedical Engineering, University of Pavia, Pavia, Italy; 5 Fightthestroke Foundation, Milan, Italy; 6 FNATC, Federazione Nazionale Associazioni Trauma Cranico, Pordenone, Italy; 7 Institute of Management, Scuola Superiore Sant’Anna, Pisa, Italy; 8 The BioRobotics Institute, Scuola Superiore Sant'Anna, Pisa, Italy; 9 Department of Excellence in Robotics & A.I., Scuola Superiore Sant'Anna, Pisa, Italy; 10 LIDER-Lab, University Scuola Superiore Sant'Anna, Pisa, Italy; 11 ITIR—Institute for Transformative Innovation Research, University of Pavia, Pavia, Italy; 12 Department of Management, Ca’ Foscari University, Venice, Italy

**Keywords:** healthcare needs, neurorehabilitation, patients' needs, survey, technological rehabilitation

## Abstract

**Introduction:**

In recent years, many robotic devices and technologies have been developed to support rehabilitation. These technologies have started penetrating clinical practice, and healthcare practitioners have started to be trained in their use, but only in selected healthcare environments. As a matter of fact, several barriers still exist for a wider dissemination of robotics-assisted rehabilitation, and little is known about the real needs of patients and their caregivers when they undertake a rehabilitation process. To address this issue a survey has been developed as a preparatory step for the effective use of robots and technologies in rehabilitation. The survey aims to identify and highlight patient's needs which are often unexpressed, enabling the co-creation of future rehabilitation solutions with patients and their caregivers.

**Methods:**

The methodology for developing the survey involves reaching out to as many people with neurological disorders as possible, understanding their needs in relation to specific functional areas, and using innovative methods such as online platforms to maximize outreach. Common functional domains for different neurological conditions were developed with a holistic view of people with disabilities, using the categories and domains defined by the new International Classification of Functioning, Disability and Health (ICF).

**Results:**

Surveys addressed to patients, caregivers of adults and of children were developed by sharing the same content and exploring the rehabilitation carried out, both traditional and technological, with particular focus on the willingness to adopt technology, if not yet integrated in the current care.

**Discussion:**

This approach aims to develop future rehabilitation strategies that utilize the increasing availability of neurorehabilitation technologies in a more effective and personalized way. This study highlights the importance of focusing on the fundamental role of addressing patients' needs in driving innovation and adoption of neurorehabilitation technologies, prior to clinical effectiveness. This is pivotal in identifying the specific desiderata, to co-create together with the end users new solutions to empower the patient-professional relationship and optimize the satisfaction related to the treatment and, as a consequence, the adherence to the intervention and its results.

## 1 Introduction

Robots and Allied Digital Technologies (RADT) have been used in rehabilitation to enhance patient care and assist healthcare professionals. RADT may enable increased rehabilitation therapy intensity, personalization of the recovery path and more active engagement of patients in therapies. Robots, on the other hand, reduce the physical stress that physiotherapists face while working with people who have motor disabilities (1).

The potential for innovative technological approaches to revolutionize rehabilitation is clear. While early RADT devices were primarily designed to increase treatment intensity, modern technologies have evolved to address a broader range of rehabilitation needs (2). These devices are now capable of rehabilitating motor functions such as movement coordination, velocity, muscle strength, balance, and walking, as well as cognitive functions like attention, memory, and executive function. Additionally, advancements in telerehabilitation and telerobotics enable remote treatment, making it possible to reach more patients and treat them simultaneously (3). These innovations have the potential to enhance rehabilitation practices, improve existing services, and provide all patients with access to advanced treatments (1). Moreover, RADT could help not only during the rehabilitation, but also when planning the rehabilitation, since they allow quantitative and objective assessment of the patients' condition and, as a consequence, the setting of specific, personalized treatment goals (4).

Despite this, the use of these technologies in clinical practice for the treatment of adults and children with neuromotor and/or cognitive impairment, remains low (5).

The first barrier regarding the implementation of RADT in clinical practice is related to the high cost. Another important limitation is sometimes the difficulty of adapting the devices to the real rehabilitative needs of patients, especially in pediatrics, and the need to train professionals, as a lot of robots, currently available, have not been designed keeping in mind their usability in clinical practice (6–8). Moreover, the lack of an adequate active engagement of stakeholders to better address the factors that contribute to technology underutilization and abandonment and facilitate better outcomes for people with disability remains a significant barrier (9). Yet, the lack of conclusive scientific evidence makes the transition to widespread rehabilitation challenging. Finally, a review of published literature suggests that many of the rehabilitation technologies do not align with the priorities of the people with disability who they are designed for, or the preferences of the health services providers who are expected to deliver them (10).

Within this framework, the Fit4MedRob initiative, an Italian collaborative project that started in December 2022, aims to overcome these barriers that currently hinder the clinical implementation of robotics in rehabilitation. The Fit4MedRob Consortium consists of 22 clinical centers and research institutes, and 3 companies. Fit4MedRob is divided into three interconnected missions: Mission 1 focuses on clinical translation and innovation, Mission 2 on the development of new biorobotic platforms and related digital technologies, and Mission 3 on basic research for future development of next-generation robots. To pursue these objectives, the project will start with pragmatic clinical trials with existing commercial devices, with the aim of providing strong scientific evidence about their effectiveness and sustainability in real-world practice. Then it will evaluate rehabilitation and personal care robots that are not yet available on the market, by capitalizing on a long-standing tradition of Italian bioengineering research. These devices could include new robotic solutions or refinements/adaptations of existing solutions to make them more compliant with actual users' needs.

To follow this workflow, the Fit4Med Consortium started with the realization of a systematic review (11) aimed to evaluate the employed methodologies for understanding patients' and healthcare practitioners' needs in the robotic rehabilitation field. This review highlighted several gaps of the current knowledge in the field, such as the absence of investigations of needs using large-scale cohorts, the non-complete representation of the most diffused neurological conditions, the small sample sizes, the limitation of assessing only some functional domains and, in most cases, the use of only a single device limiting. The literature agrees that RADT represent a significant potential in rehabilitation field, but the absence of systematic, validated methodologies does not allow to consider all end users' points of view. For this reason, the mentioned review concludes suggesting developing tailored surveys grounded in the ICF framework.

Thus, to develop these different phases of the project, the Fit4MedRob consortium agreed to start by detecting and identifying the general needs of the patients. It is widely recognized that patient care, and not only in the pediatric area, should involve engaging the patient and his family in clinical decision making. This has been confirmed to have a role in improving health outcomes, increasing satisfaction with the care experience, reducing costs and even benefit the clinician experience (12). For these reasons, in Fit4MedRob initiative, the starting point has been the detection of patients' and caregivers' rehabilitative needs and wishes with a systematic method to collect these aspects. In this framework, the methodology of surveys collecting patients' opinion on their care enables researchers to obtain information not routinely captured in other traditional ways (13).

Needs-assessment surveys have played a pivotal role in shaping the adoption and implementation of healthcare technologies across multiple clinical domains. In digital health, structured assessments of patient and caregiver needs have informed the design of telemedicine platforms, remote monitoring systems, and mobile health applications, improving usability, acceptability, and long-term adherence (14, 15). In medical device development, early-stage needs assessments have been shown to reduce technology abandonment by aligning functionality with real-world clinical workflows and user priorities (16). In assistive and rehabilitation technologies, needs-based surveys have directly influenced device customization, training strategies, and implementation models, highlighting discrepancies between technological capabilities and user expectations (17, 18). Similarly, in health information technologies, incorporating patient-reported needs has improved technology uptake by addressing perceived usefulness, burden, and contextual constraints within healthcare (19). Overall, evidence from these fields indicates that systematic needs-assessment surveys are a critical enabler of successful healthcare technology adoption, supporting user-centred design, reducing implementation failure, and fostering sustainable integration into clinical practice—principles that are increasingly recognized as essential in robotics-assisted rehabilitation.

The American Pediatric Surgery Association (APSA) define surveys as valuable tools for identifying practice preferences, understanding changes in clinical approaches, and supporting decision-making processes among healthcare professionals. When available, validated survey instruments are of course preferrable, but if this is not possible, an ad-hoc survey must be developed. Although the design, implementation and evaluation of a survey may appear to be intuitive and straightforward, multiple biases at design level may interfere with the ability of the respondent to give an accurate answer, or for the researcher to evaluate the results in a meaningful way. For this reason, the survey developers need to know all these issues and try to overcome them.

Since the systematic literature review highlighted the absence of structured and validated tools for comprehensively assessing users' needs in robotic rehabilitation (11), the Fit4MedRob consortium was prompted to idealize, design and realize an ad-hoc survey.

Starting from this background, the purpose of this work is to describe the developed survey for collecting, identifying and registering the patients' needs in the field of neurorehabilitation with the following specific objectives:

To collect and map the actual Italian landscape of the neuro-rehabilitation in terms of the different features of treatment provided (i.e., traditional and/or innovative, the setting of intervention, the treatment frequency, and related costs);
To assess patients' satisfaction with the aforementioned service provided;
To assess the access, acceptance, use of RADT in neuro-rehabilitation and, if not yet used, the desire to undergo innovative treatment

## 2 Materials and methods

### 2.1 Survey creation

Based on the systematic review (11) and the APSA guidelines for surveys (not exclusively regarding pediatric population), together with a summary of the characteristics of the surveys used in the healthcare field (20), a comprehensive guideline to be used for developing our ad-hoc survey was developed.

The first step in the survey design process is the clear definition of the research question and the selection of an appropriate study design, either descriptive or analytical. In the present study, a descriptive design was adopted, as the survey does not test a predefined hypothesis but aims to systematically collect and report data to identify general trends, as well as the incidence and prevalence of outcomes of interest.

The presented survey focuses on patients' needs, expectations, and experiences concerning both traditional rehabilitation practices and rehabilitation with advanced robotic and digital technologies. Basing on the target population which is represented by patients with a wide span of ages and different clinical pictures, existent surveys regarding patients' rehabilitative needs were analyzed: they are focused on specific diseases, in particular stroke (21–23), multiple sclerosis (24, 25) or other specific pathologies. Moreover, they involved a limited number of respondents and for the majority of questionnaires they referred to usability (26), and above all to technologies using virtual reality (25–27). The idea of this survey is instead to focus on the acceptance, barriers, satisfaction and willing to use different RADT for a wide variety of clinical conditions and by specifying the rehabilitative domain, as well as register the current traditional treatment carried out in alternative or in association to the use of RADT.

To ensure inclusivity and conceptual consistency, the International Classification of Functioning, Disability and Health (ICF) was adopted as the guiding framework. Developed by the World Health Organization (WHO), the ICF provides a standardized and internationally recognized classification system for health and disability. It supports a shift from a purely biomedical perspective to a biopsychosocial model, emphasizing the interaction between health conditions and contextual factors, including personal and environmental influences. By integrating biological, psychological, and social dimensions, the ICF enables a comprehensive understanding of functioning and disability (28, 29).

In line with the ICF framework, the survey presented in this paper has been designed to capture a multidimensional understanding of rehabilitative care and needs. The explored domains extend beyond physical and motor aspects to include social and environmental factors, reflecting the ICF's biopsychosocial approach. This allows for a more comprehensive evaluation of both traditional and technological interventions, enabling the study to provide insights into the broad spectrum of rehabilitative care. By using the ICF as a guiding framework, this survey aligns with the latest advancements in health research, offering a structured and universally applicable approach to understanding health, disability, and functioning and, in turn, patients' needs.

To make the ICF more practical for everyday use, the World Health Organization (WHO) and the ICF Research Branch (https://www.icf-research-branch.org/icf-core-sets/category/8-neurological-conditions) developed a method for creating the ICF Core Sets, based on a rigorous scientific process, including preparatory studies and input from a multidisciplinary team of experts. These Core Sets are designed to simplify the description of functioning in various contexts, such as clinical practice, by offering lists of key categories relevant to specific health conditions and care settings.

To ensure inclusivity and relevance in the developed survey, ICF Core Sets have been used, focusing on elements shared among various pathologies. The ICF Core Sets relevant to the clinical conditions encompassed by the Initiative were analyzed, drawing on resources such as those available at the ICF Research Branch (https://www.icf-research-branch.org/icf-core-sets/category/8-neurological-conditions). This involved a detailed analysis of all relevant Core Sets, including both disease-specific core sets and the generic rehabilitation ones.

In accordance with the COSMIN framework, the development of the survey was grounded in a clear definition of the construct of interest and its underlying domains. Using the ICF as the conceptual framework, all identified rehabilitation needs across different areas of functioning (such as motor, cognitive, etc) were systematically listed and assigned to overarching domains reflecting the primary construct they were intended to measure (e.g., “solving problems” within the cognitive domain).

To support content validity, a structured mapping process was conducted following the identification of individual rehabilitation needs. A dedicated table was developed to verify the relevance and representativeness of each ICF Core Set with respect to the specific clinical profiles addressed by the Fit4MedRob project: Cerebral Palsy, Multiple Sclerosis, Parkinson Disease, Post-Stroke, Acquired Brain Injury, Spinal Cord Injury, Peripheral neuromuscular diseases, Post-Oncological Surgery. When disease-specific ICF Core Sets were unavailable, the generic Core Set was applied. Throughout this process, iterative consultations with healthcare professionals and patient and parent associations were held, both in person and online, to ensure that all relevant aspects of functioning were adequately covered and that no important domains were omitted.

Informed by multidisciplinary stakeholder input, the most clinically relevant and prevalent functional domains were selected, ensuring alignment with both the target populations and the intended functional objectives of the technologies under investigation.

Subsequently, ICF categories were grouped into higher-level domains based on conceptual similarity, in line with COSMIN recommendations for domain structuring. For example, ICF categories related to walking and moving with or without assistive devices were grouped under the “mobility function” domain, capturing different modes of movement across environments. Similarly, categories describing changes in body position (e.g., sit-to-stand transitions) were included within the “postural function” domain.

Following this domain-structuring process, six main domains were identified:

mobility function;
postural function;
cognitive function;
communication function;
self-care;
upper limb function.

For each domain, an operational definition was developed to clearly specify the content covered, also accounting for age-related differences in functioning (e.g., pre-verbal communication vs. spoken language within the “communication function” domain), in accordance with COSMIN standards for transparency and interpretability.

### 2.2 Stakeholders' involvement

At various stages of the process, patients' and caregivers' associations were asked to join the process of surveys' creation. Professionals within the Fit4MedRob Consortium and patients' and caregivers' associations met in person and during online meetings, to collaborate in the co-creation of the surveys.

All individuals involved in the process were asked to review the survey to ensure clarity of the content. This was achieved by sharing a working draft of the questionnaire in a collaborative document, allowing comments, suggested modifications, and insights to be discussed and incorporated into the final version, which was newly tested in the pre-testing phase by various Fit4MedRob personnel, both clinically and non-clinically oriented (details below).

In details, the role of the representative of the associations was to ensure that all the specific needs were represented, by eventually integrate the ICF Core sets, moreover they checked the language of the survey to be inclusive and respectful. When the survey was developed as a draft, they tested it with the aim of verified its feasibility in terms of time-consuming effort and this contributes to the ramification architecture of the survey (described below) for saving time and focus on the most important rehabilitation domains. Finally, this shared process helped in raising awareness of the importance of data collection for the further disseminative phase.

In summary, the development of the survey followed a structured workflow informed by the Donabedian criteria (Structure-Process-Outcome) and guided by COSMIN (Consensus-based Standards for the selection of health Measurement INstruments) criteria and in particular:

*Structure:* According to the Donabedian framework, the structural phase involved defining the resources, expertise and context required for survey development. An interdisciplinary team composed of clinicians, rehabilitation engineers, and researchers collaboratively identified the key domains of interest. Existing literature on technology adoption, rehabilitation robotics and user needs analysis was reviewed to ensure that the survey covered relevant constructs.
*Process:* In line with COSMIN recommendations, we adopted a transparent, iterative process to ensure content validity. Item generation was based on literature synthesis, expert consultation, and preliminary discussions with clinicians and end-users. Draft items were reviewed by a panel of experts for clarity, relevance and comprehensiveness. Interviews and a pilot test with a small sample of professionals working in paediatric rehabilitation were conducted to assess item interpretation, acceptability and feasibility. Feedback from this stage informed refinement of wording, ordering and response formats.
*Outcomes:* The outcome level of the Donabedian model guided the definition of what the survey aims to measure: priorities, perceived needs, satisfaction with current technologies and willingness to adopt new tools in clinical practice. Following COSMIN principles, we ensured that the survey captures these constructs in a way that is meaningful, interpretable and aligned with clinical decision-making. The final version of the survey is designed to support future psychometric evaluation (e.g., reliability and construct validity) in larger samples.

### 2.3 Survey description

To allow respondents to freely express their opinions the survey was anonymous. On the other hand, demographic characteristics are pivotal in the data analysis to stratify respondents in homogeneous categories; for this reason, a first general section has been planned with some generic questions about individual characteristics, such as the age range and referral clinical center (data that cannot lead back to the patient's identity).

Then, following the ICF framework, specific needs associated with various functional domains were categorized, aligning each need with its overarching domain and grouping questions about the same topic together, as suggested by the literature (30):

**Mobility.** It aims to define different mobility situations related to various movement conditions, such as walking forward, backward, or laterally, moving on different surfaces (indoors and outdoors), navigating or overcoming obstacles, climbing and descending stairs, running, and jumping. It also includes movements using specific devices designed to assist with movement, such as manual or electronic wheelchairs, walkers, and canes.
**Postural function.** It focuses on functions related to assuming, maintaining, and changing different postures, including lying down (supine), lying on one's side, sitting, standing, kneeling, and others.
**Cognitive Functions.** This addresses cognitive and neuropsychological functions involved in behaviors such as, problem-solving by identifying and analyzing issues, decision-making between options and evaluating the effects of those choices (e.g., selecting an item to purchase or deciding between different tasks to complete), coordinating simple or complex actions to plan, manage, and accomplish daily tasks, including time management and organizing activities throughout the day.
**Communication.** This means both verbal and non-verbal communication functions, which include understanding the literal and implied meanings of spoken messages, such as distinguishing between factual statements and idiomatic expressions, producing words or sentences of varying lengths to express facts or tell stories, initiating, sustaining, and concluding conversations with one or more people, using devices, techniques, or other means to communicate, such as calling someone on the phone, utilizing telecommunication devices, writing technologies, or speech assistive technologies, employing gestures, symbols, and drawings to convey messages, such as shaking one's head to indicate disagreement or drawing to express a complex idea. This also involves producing or using gestures, signs, symbols, and images for communication.
**Self-Care.** It refers to functions involved in daily routines and self-care tasks, such as preparing food, eating, pouring drinks, dressing, washing, and taking care of personal hygiene.
**Upper Limb and Hand Use.** This refers to functions related to the use of the hands and arms, including, coordinated actions such as handling, picking up, manipulating, and releasing objects with the hands, fingers, and thumbs, moving or manipulating objects with hands and arms, such as turning door handles, throwing or catching objects, or pushing and pulling items.

For each domain, an explicative definition has been further formulated by integrating the specific description of ICF Core Sets, also considering the specific potential differences among ages (e.g., the specific needs related to pre-talking and speaking in the “communication function” for very young children). By grouping the ICF items, functional domains of included conditions, considered to be the most represented ones, were identified.

Each of the six domains was investigated by a 5-point Likert scale analysis, to gather the following key parameters:

**Independence**: assessment of patient's independence and need for assistance or assistive devices, based on common classification systems like the Gross Motor Function Classification System (for movement abilities) and the Manual Ability Classification System (for hand use).
**Impact on life quality:** quantification of the impairment impact on the patients' quality of life.
**Current rehabilitation treatment** (referred to the last 12 months), more in details:
  kind of treatment (traditional and technological, with related dosage)
  setting (in-clinic or at home)
  satisfaction and, in case of absence of the treatment, the will and the need of the treatment

Given the importance of detecting possible financial toxicity for patients and their families, a final section of the survey was dedicated to gathering data on rehabilitation costs. Starting with a yes/no question on the provision of rehabilitation (both traditional and technological) specific costs were investigated. This included expenses for devices (e.g., walking aids or rental of assistive devices), home adaptations (e.g., ramps), special food (supplements), transportation, hired assistance (both medical professionals like nurses and non-medical help like babysitters), and income loss due to the need for caregivers to assist patients. For each question, different cost ranges have been proposed, based on the general costs traditionally described for rehabilitation. In fact, health-related cost data can be collected through direct measurement approaches, including structured surveys and self-reported questionnaires, which are commonly used when administrative records are unavailable or incomplete. Surveys capture resource use by asking respondents to report on service utilization and associated out-of-pocket expenditures, with appropriate recall periods (often 6–12 months) to balance comprehensiveness and recall accuracy. Such methodologies, along with clear definitions of cost elements and standardised data collection procedures, are recommended for economic evaluations and burden-of-illness studies (31, 32).

Then, in the final part of the questionnaire, at the end of all six domains and the economic section describe d below, subjects are asked to rank the domains in order of importance, i.e., to list them hierarchically from a subjective perspective.

### 2.4 Survey versions

Guided by the ICF framework, which emphasizes inclusivity across biological, social, and environmental domains, and considering that the target population comprises both children and adults with disabilities, different versions of the survey were developed. This allowed to assess the use of rehabilit ation technology across different age ranges and without excluding any group of participants with sensory, motor, and/or cognitive disability. More precisely, the following three surveys have been created:

Survey for collaborative adult patients, able to answer independently;
Survey for caregivers of non-collaborative adult patients;
Survey for caregivers of children with disabilities.

They share the same overall structure, while they are adapted to the different populations by phrasing questions differently depending on the respondent. In surveys 2 and 3, caregivers were asked to represent and express the needs of the patient.

The adaptations have been made with the purpose of maximizing the survey's success and ensure respondents provide honest and meaningful answers. In particular, based on the principle of clarity and precision (33), vague terms like “usually” or “few” were avoided, opting instead for phrases that are temporally specific, such as “once a week” or “at least once a year”. Furthermore, visual aids were incorporated to clarify questions and enhance comprehension. In addition, an accessible language has been used: clear and widely understood terms were selected, considering their frequency of use in everyday language, to ensure accessibility and inclusivity. By designing the survey this way, the ICF's principles were followed, making sure it captured the views of the different characters involved in rehabilitation, without leaving anyone out.

Regarding the formulation of the questions, a 5-point Likert scale was selected to collect precise and detailed data (13).

Then, with the aim of avoiding excessive length of the survey, questions were structured with a ramification logic. Although all questions were mandatory, many were displayed only if specific conditions were met. Specifically, following the consent request, the first section collected general demographic information using closed-ended questions to ensure respondent anonymization while still capturing key characteristics such as age range. The collected information included age range, gender, clinical condition (selected from a predefined list with an “other” option for further specification), rehabilitation setting in which the child was receiving care, region of residence within Italy, and the child's reported familiarity with technology.

For each of the six domains, respondents were asked to rate the child's level of independence and the impact of limitations within that domain using 5-point scales. Subsequently, current rehabilitation treatments received during the previous year were investigated. First, traditional rehabilitation treatments (i.e., without the use of technological solutions) were assessed using five response options: (i) no, and this is acceptable; (ii) no, but the respondent would like to; (iii) partially (no more than once per week); (iv) regularly (twice per week or more); and (v) extensively (intensive treatment). For the latter three options, respondents were also asked to report their level of satisfaction and the treatment setting (inpatient, day hospital, or home-based). For each setting, response options included: (i) no, and this is acceptable; (ii) no, but the respondent would like to; (iii) yes, but the respondent is not satisfied; and (iv) yes, and the respondent is satisfied.

In cases where no traditional rehabilitation treatment was reported, the same set of questions was applied to technology-based rehabilitation. When a technological intervention was ongoing, respondents were additionally asked to specify the name(s) of the devices used. At the end of each domain section, and whenever a technological treatment was present, respondents were asked about their willingness to receive more information about rehabilitation technologies.

This conditional logic was applied consistently across all six domains, so that questions were displayed only when relevant (i.e., in the presence of ongoing treatment), thereby optimizing questionnaire completion time and respondent burden. This structure, developed with valuable input from parent associations, was designed to collect detailed, domain-specific information while minimizing questionnaire length and effort.

After completing the six domains, respondents were asked to report the economic impact of rehabilitation treatments over the previous year, using five cost ranges (0; <€500; €501–€2,000; €2,001–€5,000; >€5,000).

Finally, in the concluding section, respondents were asked to rank the six domains according to perceived priority, and an open-text field was provided to collect additional comments.

The survey is structured across multiple pages, with a progress bar displayed to inform respondents of their completion status.

**Figure 1:**
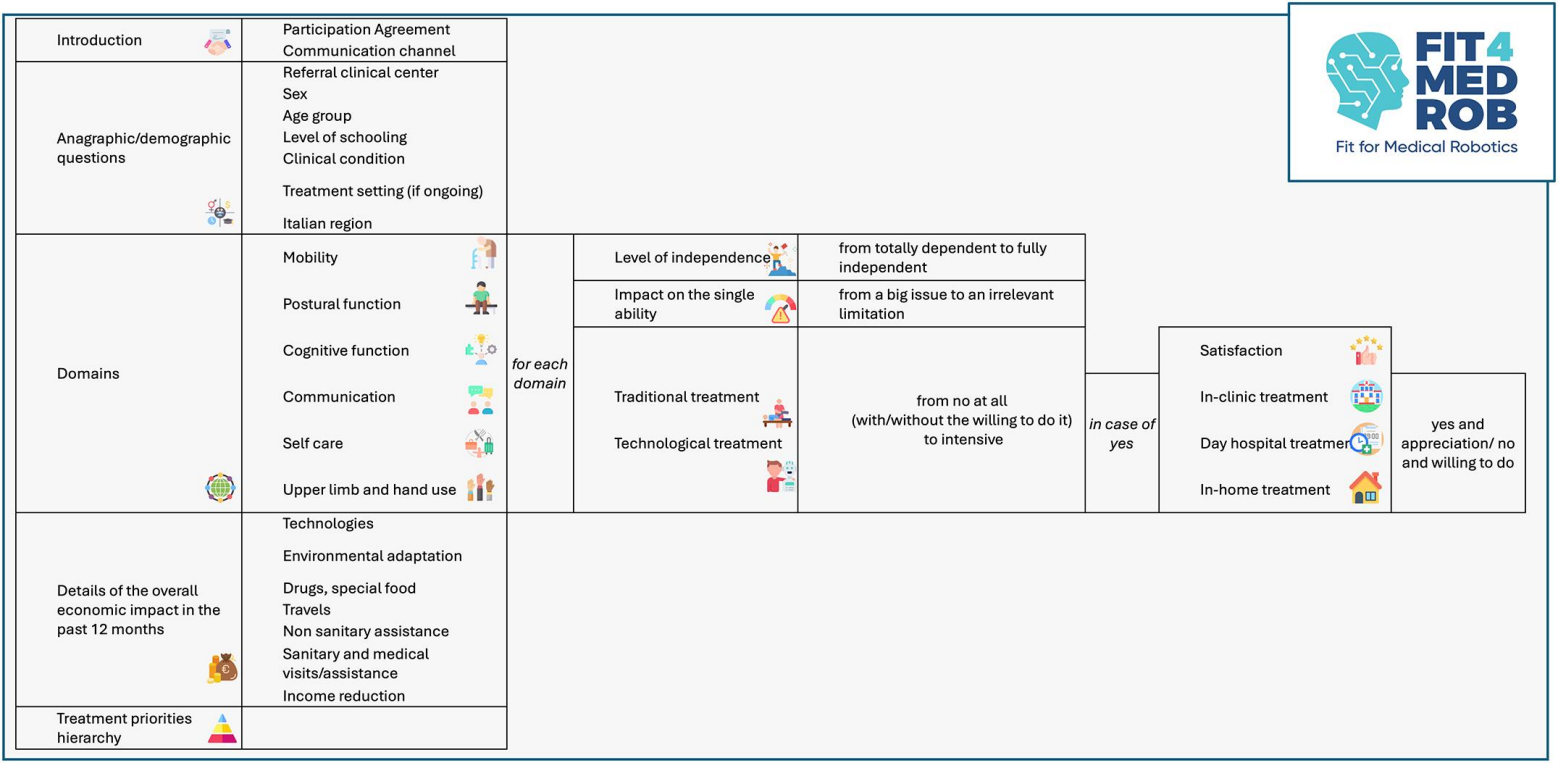
Survey structure and assessed domains. The survey overall structure is presented: it includes a first introductory part where consent is collected, then some non-identifiable demographic variables are collected using categorical ranges. The central part is represented by six ICF-based functional domains (mobility, postural function, cognitive function, communication, self-care, and upper limb and hand use), and each one is evaluated in terms of independence, impact on quality of life, and rehabilitation treatment characteristics (type, setting, and satisfaction) for both the traditional and the technological one; in particular. The survey finally investigates rehabilitation-related costs over the last 12 months and concludes with a hierarchical ranking of domain importance from patient's perspective. “FIT 4 MED ROB” logo reproduced with permission from Fit for Medical Robotics Foundation (https://www.fit4medrob.it/). Icons from Flaticon (https://www.flaticon.com/), licensed under Premium License.

The survey structure is summarized in Figure 1.

As suggested by existing guidelines, the surveys were pre-tested with various Fit4MedRob personnel, both clinically oriented (such as physicians or therapists) and non-clinically oriented (such as engineers). Specifically, the surveys were tested by 5 physicians, 8 physical therapists, and 4 biomedical engineers. Personnel was selected to represent a range of backgrounds and expertise. Additionally, similar feedback was sought from patients' and caregivers' associations regarding the readability, length, and potential misunderstandings of survey contents.

As a result, minor revisions to the wording were implemented, including correction of typographical errors and rephrasing to enhance clarity. In addition, the completion time was tested and estimated to range from approximately 20 to 35 min, depending on the branching logic activated by respondents' answers.

### 2.5 Survey dissemination

Among the potential approaches to administering a survey, such as face-to-face interviews, telephone interviews, self-completion questionnaires and computer-assisted methods (20, 34), there is no clear evidence that responses to sensitive questions differ between the various administration methods and no single method consistently outperforms all others. In the described Initiative, for a self-completion approach, utilizing an internet-based tool (Microsoft Forms) was chosen to ensure accessibility for all users via their personal smartphone, computer or tablet. The use of technologies which includes websites and smartphones has been suggested as increasing engagement of patients in goal-setting and definition of the rehabilitation content (35). This also facilitates the completion of a comprehensive survey avoiding taking a significant time and guarantees more freedom in disclosing personal feelings and impressions. An internet-based tool allows choosing a convenient time for starting and completing the survey and avoiding the challenge of obtaining responses on sensitive topics in presence of an interviewer. Thus, an online tool also facilitates the dissemination of the survey. In fact, distributing survey links via social media accounts of individual users and organized e-groups with an interest in specific health issues may increase the engagement and accuracy of responses (36). Institutional channels of all the clinical centers involved in the Fit4MedRob Consortium and of the several patients' and caregivers' associations were identified. Additionally, social media of all the clinical centers involved in the Fit4MedRob Consortium were used and in-person events (such as the Fit4MedRob conferences showing ad-hoc QR codes) were organized, to facilitate engagement.

## 3 Discussion

Despite the growing interest among researchers, as evidenced by a substantial body of scientific output and the rapid expansion of RADT solutions for rehabilitation, the literature on the effectiveness of technology remains limited. The majority of studies, including meta-analyses, primarily focus on stroke patients (6, 8, 22) and the results are often inconclusive. This is due to various factors, particularly the considerable heterogeneity of studies in terms of treatment duration, session frequency, and specific modalities as stated also by the CICERONE network (37, 38). Additionally, the small sample sizes in many studies pose a significant challenge to evaluating their overall effectiveness (23). Research on other neurological conditions, such as traumatic brain injury, cerebral palsy, and spinal cord injury, is even more scarce and typically involves small patient cohorts.

A significant reason for lack of evidence about the use of technology in rehabilitation could be the fact that the different devices and protocols have been developed considering only the point of view of one type of end users, often avoiding the co-design and the co-creation with the patients (including the caregivers and the representation such as the associations).

To the best of our knowledge, this is the first methodological approach which, by means of a survey created ad-hoc, specifically investigates the use of robots and technology in the rehabilitation of adults and children with sensory, motor, and/or cognitive disorders from the viewpoint of direct end users. Unlike other studies that rely on clinicians or researchers as primary respondents, this tool empowers those who are directly involved in the rehabilitation process—patients or caregivers—to share their insights. This approach allows for a richer, more nuanced understanding of how technological tools are utilized in real-world scenarios, ensuring that the outcomes and recommendations align closely with the actual needs and preferences of users. The survey also plays a pivotal role in advancing the objectives of the Fit4MedRob initiative. By gathering data directly from end users, it provides valuable insights into the effectiveness, usability, and practical applications of neurorehabilitation tools. These findings not only support the evaluation of existing instruments but also inform the development of future technologies, ensuring they are tailored to the diverse needs of individuals undergoing rehabilitation.

The survey design is deeply rooted in the International Classification of Functioning, Disability and Health (ICF) framework, which advocates for a holistic approach to rehabilitation. This framework underscores the importance of addressing multiple dimensions of an individual's well-being. In line with this, the survey goes beyond focusing solely on neurological and motor impairments. It considers a broader spectrum of life aspects, such as socialization, participation in community activities, and integration into various settings, including home, school, and work environments. By capturing these diverse domains, the survey provides a comprehensive understanding of the factors that contribute to meaningful rehabilitation outcomes. One of the core principles guiding the survey is the emphasis on health rather than illness. This perspective shifts the focus from managing symptoms to enhancing the overall quality of life. By exploring the needs of individuals as people first—rather than solely as patients—the survey aims to uncover opportunities to support their independence, engagement, and participation in everyday activities. This paradigm shift aligns with modern rehabilitation practices, which prioritize empowering individuals to lead fulfilling lives.

A standout feature of the questionnaire is its inclusivity. It has been meticulously designed to accommodate a diverse range of respondents, including individuals with varying conditions, diseases, or disabilities, as well as caregivers and adults from different backgrounds. This inclusivity ensures that no perspective is overlooked, allowing for a more comprehensive understanding of needs across a broad spectrum of users. By doing so, the questionnaire captures the diverse experiences of participants.

The questionnaire incorporates multiple functional domains, reflecting the complexity of rehabilitation needs. These domains span physical performance, cognitive functions, and ability in daily living, providing a multidimensional view of the challenges and opportunities faced by individuals in rehabilitation.

This is a powerful methodological approach in which a tool has been developed, enabling a real inclusion of the end users (patients) in the definition of shared needs and in particular in setting rehabilitative goals and this has been demonstrated to have an energizing function whereby participation in the goal-setting process increases performance, and persistence to achieve specific goal (39).

By combining inclusivity, a health-centered approach, and adherence to the ICF framework, this survey sets a new standard for understanding the role of technology in neurorehabilitation. Its emphasis on the lived experiences of end users ensures that the insights generated are both practical and transformative, paving the way for more effective and personalized rehabilitation solutions. Eventually, incorporating questions related to financial toxicity, the survey allows capturing useful information for sustainability and health technology assessment studies.

This proposed survey method could serve as a foundation for future research aimed at promoting the adoption and integration of digital health technologies in motor rehabilitation, both in the pediatric and adult field. Before extending this tool to other contexts, a formal validation plan should be implemented. Accordingly, the current pilot testing phase should be followed by an assessment of intra- and inter-rater reliability, conducted on a selected subgroup of stakeholders with varying levels of experience with technology, who would complete the survey in a non-anonymized manner specifically for validation purposes. Then, once the psychometric properties will be demonstrated, a wider adoption could be planned.

This study has several limitations that should be acknowledged. First, the use of an online, self-administered survey may have introduced selection bias, as individuals with greater digital literacy, higher engagement, or stronger interest in technology-assisted rehabilitation may have been more likely to participate. Second, some information related to rehabilitation pathways and costs relies on the Long-Term Care Insurance System as a proxy, which may not fully reflect the heterogeneity and complexity of individual care trajectories and is therefore inherently subject to bias; Italy's Long-Term Care relies heavily on family, especially children, who provide informal support, reducing demand for private insurance but creating high personal costs. Third, the inclusion of a heterogeneous population with multiple neurological conditions limits the possibility of drawing disease-specific conclusions and may reduce comparability across subgroups since the sample presumably present different sizes. Finally, data were self-reported and referred retrospectively to experiences over the previous 12 months, which exposes the results to potential recall bias. Despite these limitations, the survey provides a structured and patient-centred overview of perceived needs, priorities, and experiences with rehabilitation technologies, which can inform future validation studies and targeted research.

Since the surveys are based on ICF, so an international framework, and also translated in English, they could be easily adopted by researchers of other countries than Italy. Such studies might explore strategies to enhance user engagement, assess the effectiveness of these technologies in improving motor outcomes, and identify potential barriers and facilitators to their implementation in clinical and home settings in different landscapes. When applying this methodological approach in other countries, which may have different healthcare systems, technological infrastructures, and reimbursement models, careful attention must be given to adapting the survey to the local context. The key point is that it should still capture the needs in terms of priorities, satisfaction, and willingness to adopt technologies. The greatest challenge, however, will be addressing these needs in a way that is tailored to the specific context.

Anyway, by leveraging this model, researchers can contribute to the development of evidence-based approaches that support the widespread and effective use of digital health solutions in rehabilitation.

## Data Availability

The original contributions presented in the study are included in the article/Supplementary Material, further inquiries can be directed to the corresponding authors.

## Mission 1 Fit4MedRob consortium

Corpina, Flavio; Ferlazzo, Ennio; Gasperini, Giulio; Specchia, Alessandro; Molteni, Franco; Azzollini, Valentina; Dalise, Stefania; Chisari, Carmelo; Palomba, Angela; Gnasso, Rossana; Ruosi, Carlo; Zamboni, Giovanna; Mandrioli, Jessica; Benuzzi, Francesca; Gennari, Francesca; Pittaluga, Matteo; Schenone, Angelo; Hamedani, Mehrnaz; Diella, Eleonora; Storm, Fabio; Biffi, Emilia; Aiello, Alessia; Tacchino, Chiara Maria; Gallotta, Matteo; Piras, Rachele; Lunetta, Christian; Fundarò, Cira; Pavese, Chiara; Nardone, Antonio; Trane, Stefania; Minafra, Brigida; Fiore, Pietro; Antonacci, Carla; Carnevale, Arianna; Longo, Giuseppe Umile; Nicotra, Roberta; Signorini, Sabrina; Campese, Ilaria; Martinis, Luca; De Icco, Roberto; D'Oronzio, Stefano Giuseppe; Castagnoli, Chiara; Piazzini, Michele; Ciapetti, Tommaso; Cecchi, Francesca; Mosca, Rita.

## References

[1] e Siqueira TB Parraça J Sousa JP. Available rehabilitation technology with the potential to be incorporated into the clinical practice of physiotherapists: a systematic review. Health Sci Rep. (2024) 7(4):e1920. 10.1002/hsr2.1920 38605728 PMC11007654

[2] Laut J Porfiri M Raghavan P. The present and future of robotic technology in rehabilitation. Curr Phys Med Rehabil Rep. (2016) 4(4):312–9. 10.1007/s40141-016-0139-0 28603663 PMC5461931

[3] Galea MD. Telemedicine in rehabilitation. Phys Med Rehabil Clin N Am. (2019) 30(2):473–83. 10.1016/j.pmr.2018.12.002 30954160

[4] van Vliet P Wing AM. A new challenge–robotics in the rehabilitation of the neurologically motor impaired. Phys Ther. (1991) 71(1):39–47. 10.1093/ptj/71.1.39 1984250

[5] Ouendi N Hubaut R Pelayo S Anceaux F Wallard L. The rehabilitation robot: factors influencing its use, advantages and limitations in clinical rehabilitation. Disabil Rehabil Assist Technol. (2024) 19(3):546–57. 10.1080/17483107.2022.2107095 35921160

[6] Babaiasl M Mahdioun SH Jaryani P Yazdani M. A review of technological and clinical aspects of robot-aided rehabilitation of upper-extremity after stroke. Disabil Rehabil Assist Technol. (2016) 11(4):263–80. 10.3109/17483107.2014.1002539 25600057

[7] Li L Tyson S Weightman A. Professionals’ views and experiences of using rehabilitation robotics with stroke survivors: a mixed methods survey. Front Med Technol. (2021) 3:780090. 10.3389/fmedt.2021.780090 35047969 PMC8757825

[8] Li L Fu Q Tyson S Preston N Weightman A. A scoping review of design requirements for a home-based upper limb rehabilitation robot for stroke. Top Stroke Rehabil. (2022) 29(6):449–63. 10.1080/10749357.2021.1943797 34281494

[9] Mitchell J Shirota C Clanchy K. Factors that influence the adoption of rehabilitation technologies: a multi-disciplinary qualitative exploration. J Neuroeng Rehabil. (2023) 20(1):80. 10.1186/s12984-023-01194-9 37340496 PMC10280872

[10] Musselman KE Shah M Zariffa J. Rehabilitation technologies and interventions for individuals with spinal cord injury: translational potential of current trends. J Neuroeng Rehabil. (2018) 15(1):40. 10.1186/s12984-018-0386-7 29769082 PMC5956557

[11] Fasano A Mauro MC Beani E Nicora G Germanotta M Falchini F Towards the identification of patients’ needs for promoting robotics and allied digital technologies in rehabilitation: a systematic review. Healthc Basel Switz. (2025) 13(7):828. 10.3390/healthcare13070828 PMC11988541 40218126

[12] Bombard Y Baker GR Orlando E Fancott C Bhatia P Casalino S Engaging patients to improve quality of care: a systematic review. Implement Sci. (2018) 13(1):98. 10.1186/s13012-018-0784-z 30045735 PMC6060529

[13] Kelley-Quon LI. Surveys: merging qualitative and quantitative research methods. Semin Pediatr Surg. (2018) 27(6):361–6. 10.1053/j.sempedsurg.2018.10.007 30473040

[14] Gemert-Pijnen Jv Nijland N Limburg Mv Ossebaard HC Kelders SM Eysenbach G A holistic framework to improve the uptake and impact of eHealth technologies. J Med Internet Res. (2011) 13(4):e1672. 10.2196/jmir.1672 PMC3278097 22155738

[15] Greenhalgh T Robert G Macfarlane F Bate P Kyriakidou O. Diffusion of innovations in service organizations: systematic review and recommendations. Milbank Q. (2004) 82(4):581–629. 10.1111/j.0887-378X.2004.00325.x 15595944 PMC2690184

[16] McGinn CA Gagnon MP Shaw N Sicotte C Mathieu L Leduc Y Users’ perspectives of key factors to implementing electronic health records in Canada: a Delphi study. BMC Med Inform Decis Mak. (2012) 12(1):105. 10.1186/1472-6947-12-105 22967231 PMC3470948

[17] Scherer M. 29 Assistive Technology for Older Adults: From Bedside to Curbside (2017). p. 591–604.

[18] Demers L Fuhrer MJ Jutai J Lenker J Depa M De Ruyter F. A conceptual framework of outcomes for caregivers of assistive technology users. Am J Phys Med Rehabil. (2009) 88(8):645–55. 10.1097/PHM.0b013e3181ae0e70 19620830

[19] Holden RJ Karsh BT. The technology acceptance model: its past and its future in health care. J Biomed Inform. (2010) 43(1):159–72. 10.1016/j.jbi.2009.07.002 19615467 PMC2814963

[20] McColl E Jacoby A Thomas L Soutter J Bamford C Steen N Design and use of questionnaires: a review of best practice applicable to surveys of health service staff and patients. Health Technol Assess Winch Engl. (2001) 5(31):1–256. 10.3310/hta5310 11809125

[21] Sauerzopf L Luft A Maeusli V Klamroth-Marganska V Sy M Spiess MR. Technology use for home-based stroke rehabilitation in Switzerland from the perspectives of persons living with stroke, informal caregivers, and therapists: qualitative interview and focus group study. JMIR Rehabil Assist Technol. (2024) 11:e59781. 10.2196/59781 39024576 PMC11294768

[22] Luo Y Yang J Zhang Y. Development and validation of a patient-reported outcome measure for stroke patients. Health Qual Life Outcomes. (2015) 13:53. 10.1186/s12955-015-0246-0 25953508 PMC4489208

[23] Guillén-Climent S Garzo A Muñoz-Alcaraz MN Casado-Adam P Arcas-Ruiz-Ruano J Mejías-Ruiz M A usability study in patients with stroke using MERLIN, a robotic system based on serious games for upper limb rehabilitation in the home setting. J NeuroEngineering Rehabil. (2021) 18(1):41. 10.1186/s12984-021-00837-z PMC7901008 33622344

[24] Meca-Lallana V Prefasi D Alabarcez W Hernández T García-Vaz F Portaña A A pilot study to explore patient satisfaction with a virtual rehabilitation program in multiple sclerosis: the RehabVR study protocol. Front Neurol. (2020) 11:900. 10.3389/fneur.2020.00900 33162924 PMC7580492

[25] Lozano-Quilis JA Gil-Gómez H Gil-Gómez JA Albiol-Pérez S Palacios-Navarro G Fardoun HM Virtual rehabilitation for multiple sclerosis using a kinect-based system: randomized controlled trial. JMIR Serious Games. (2014) 2(2):e12. 10.2196/games.2933 25654242 PMC4307818

[26] Gil-Gómez JA Manzano-Hernández P Albiol-Pérez S Aula-Valero C Gil-Gómez H Lozano-Quilis JA. USEQ: a short questionnaire for satisfaction evaluation of virtual rehabilitation systems. Sensors. (2017) 17(7):1589. 10.3390/s17071589 28686174 PMC5539644

[27] Iosa M Aydin M Candelise C Coda N Morone G Antonucci G The michelangelo effect: art improves the performance in a virtual reality task developed for upper limb neurorehabilitation. Front Psychol. (2021) 11:611956. 10.3389/fpsyg.2020.611956 33488478 PMC7817887

[28] Vargus-Adams JN Majnemer A. International classification of functioning, disability and health (ICF) as a framework for change: revolutionizing rehabilitation. J Child Neurol. (2014) 29(8):1030–5. 10.1177/0883073814533595 24850572

[29] Leonardi M Lee H Kostanjsek N Fornari A Raggi A Martinuzzi A 20 years of ICF—international classification of functioning, disability and health: uses and applications around the world. Int J Environ Res Public Health. (2022) 19(18):11321. 10.3390/ijerph191811321 36141593 PMC9517056

[30] Burns KEA Duffett M Kho ME Meade MO Adhikari NKJ Sinuff T A guide for the design and conduct of self-administered surveys of clinicians. CMAJ. (2008) 179(3):245–52. 10.1503/cmaj.080372 18663204 PMC2474876

[31] Smith KA Rudmik L. Cost collection and analysis for health economic evaluation. Otolaryngol–Head Neck Surg Off J Am Acad Otolaryngol-Head Neck Surg. (2013) 149(2):192–9. 10.1177/0194599813487850 23641023

[32] Jabakhanji SB Sorensen J Valentelyte G Burke LA McElroy B Murphy A. Assessing direct healthcare costs when restricted to self-reported data: a scoping review. Health Econ Rev. (2021) 11(1):35. 10.1186/s13561-021-00330-2 34529165 PMC8444520

[33] Goldin AB LaRiviere C Arca MJ Cassidy L Abdullah F Lee SL Guidelines for surveys of the American pediatric surgical association. J Pediatr Surg. (2011) 46(10):2012–7. 10.1016/j.jpedsurg.2011.05.016 22008342

[34] Stone DH. Design a questionnaire. Br Med J. (1993) 307(6914):1264–6. 10.1136/bmj.307.6914.1264 8281062 PMC1679392

[35] Dicianno BE Henderson G Parmanto B. Design of Mobile health tools to promote goal achievement in self-management tasks. JMIR MHealth UHealth. (2017) 5(7):e103. 10.2196/mhealth.7335 28739558 PMC5547247

[36] Savard I Kilpatrick K. Tailoring research recruitment strategies to survey harder-to-reach populations: a discussion paper. J Adv Nurs. (2022) 78(4):968–78. 10.1111/jan.15156 35084799

[37] Turolla A Kiper P Mazzarotto D Cecchi F Colucci M D’Avenio G Reference theories and future perspectives on robot-assisted rehabilitation in people with neurological conditions: a scoping review and recommendations from the Italian consensus conference on robotics in neurorehabilitation (CICERONE). NeuroRehabilitation. (2022) 51(4):681–91. 10.3233/NRE-220160 36530100

[38] BiblioSan. Robot-Assisted Rehabilitation for Children with Neurological Disabilities: Results of the Italian Consensus Conference CICERONE—PubMed. Available online at: https://pubmed-ncbi-nlm-nih-gov.bibliosan.idm.oclc.org/36530098/ (Accessed April 28, 2025). 10.3233/NRE-220036 36530098

[39] Locke EA Latham GP. Building a practically useful theory of goal setting and task motivation. A 35-year odyssey. Am Psychol. (2002) 57(9):705–17. 10.1037//0003-066x.57.9.705 12237980

